# Supplementary material for: Association of Blood Metabolomics Biomarkers with Brain Metabolites and Patient-Reported Outcomes as a New Approach in Individualized Diagnosis of Schizophrenia
Source: Int J Mol Sci. 2024 Feb 15;25(4):2294. doi: 10.3390/ijms25042294 (PMC10888632; doi:10.3390/ijms25042294)
Supplement: Supplementary file 1 [file ijms-25-02294-s001.zip › ijms-2842902-submit-supplementary.pdf]

Additional analysis to the results regarding article:

Bryll, A.; Krzyściak, W.; Karcz, P.; Pilecki, M.; Śmierciak, N.; Szwajca, M.; Skalniak, A.; Popiela, T.J. Determinants of Schizophrenia Endophenotypes Based on Neuroimaging and Biochemical Parameters. *Biomedicines* **2021**, *9*, 372. https://doi.org/10.3390/biomedicines9040372.

Attention was drawn to the fact that the change in the total score of the scale (T) had a strong relationship with the level of glutamic acid in the blood. The obtained results were confirmed by a cluster analysis (unpublished own results). Two clusters of people were identified, i.e. with a low change in T score (M = 37.75; SD = 8.73) and a high one (M = 64.92; SD = 6.14). In the cluster of people with a low change in T scores (n = 24 - 64.9%), the level of glutamic acid turned out to be statistically significantly higher compared to people with a high change in T score, U = 49; p = 0.003 (n = 13 - 35.1%).

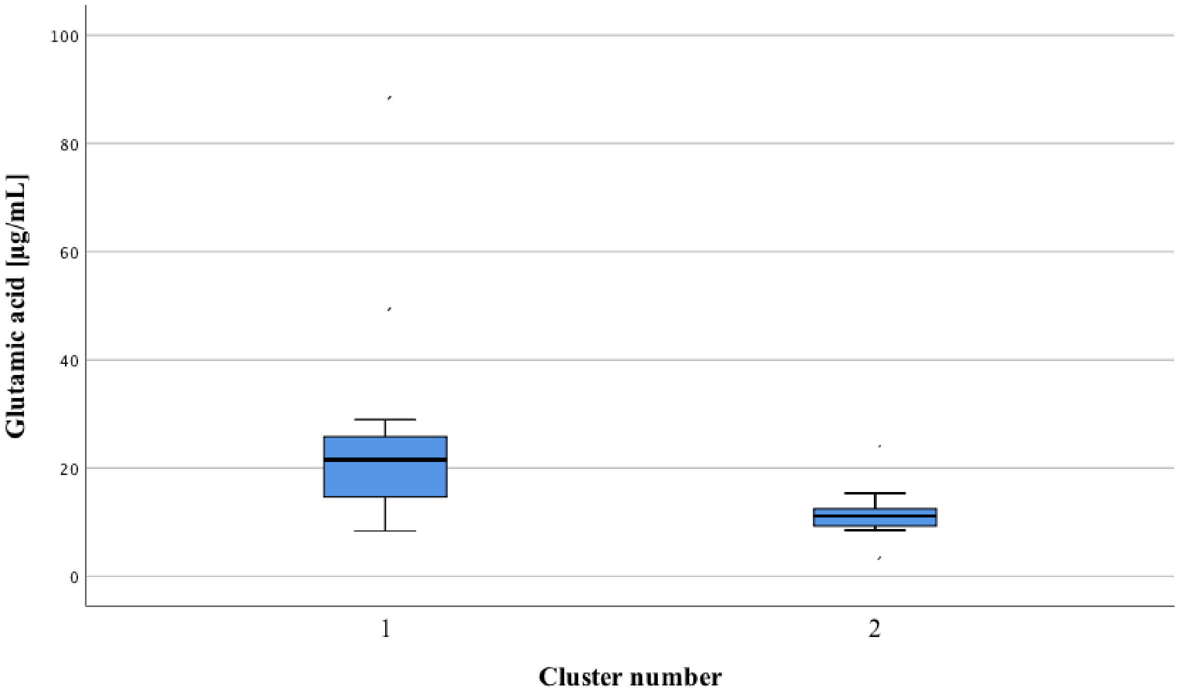

Figure S1. The level of glutamic acid in isolated clusters of people based on the change in the total T score.

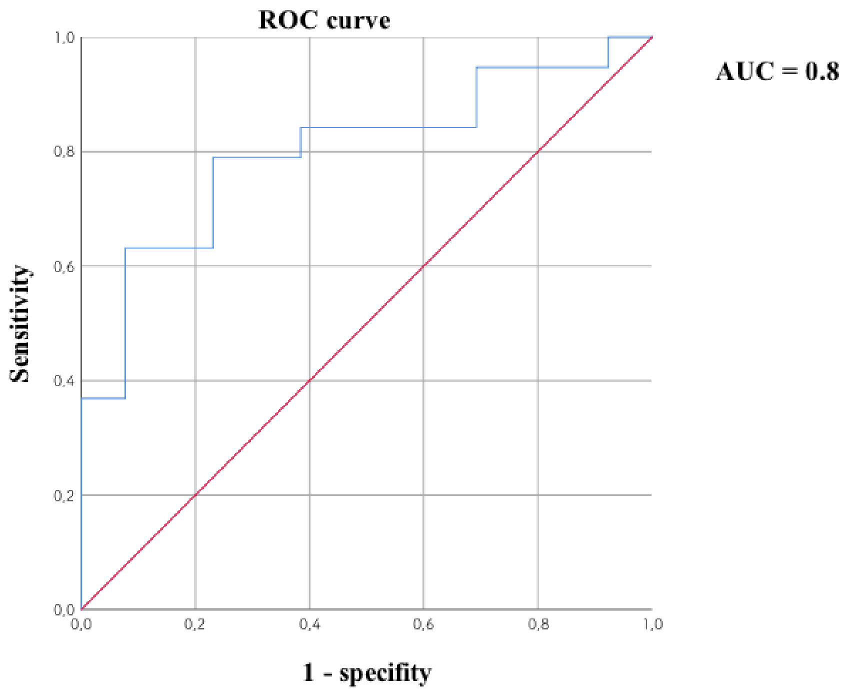

Figure S2. ROC curve for glutamic acid levels (vs change in T score).
